# Management of pregnancy and outcomes in a woman with mannose-6-phosphate isomerase deficiency (MPI-CDG)

**DOI:** 10.1016/j.ymgmr.2026.101329

**Published:** 2026-06-17

**Authors:** Jia Hui Megan Loh, May Oo Khin, John Bassett, Amal Mighell, Sarah Jowett, Sulleman Moreea, Alison Woodall, Priscilla Sia, Afifah Hussain, Amy Hufton, Karolina M. Stepien

**Affiliations:** a The School of Medicine, Manchester Academic Health Sciences Centre, Manchester University, UK; b Adult Inherited Metabolic Diseases, Salford Care Organization, Northern Care Alliance NHS Foundation Trust, Salford, UK; c Obstetrics and Gynaecology Department, Bradford Teaching Hospitals NHS Foundation Trust, Bradford, UK; d Gastroenterology Department, Bradford Teaching Hospitals NHS Foundation Trust, Bradford, UK; e Urgent Ambulatory Care, Bradford Teaching Hospitals NHS Foundation Trust, Bradford, UK

**Keywords:** Congenital glycosylation defects, CDG Ib, MPI-CDG, Pregnancy, Outcomes

## Abstract

Congenital Disorder of Glycosylation type Ib (MPI-CDG) is a rare autosomal recessive metabolic disorder caused by deficiency of mannose-6-phosphate isomerase, resulting in impaired N-glycosylation. Unlike other CDG subtypes, MPI-CDG is not associated with neurodevelopmental impairment and is effectively treated with oral D-mannose supplementation. However, evidence guiding management during pregnancy is scarce, and D-mannose is generally avoided due to limited safety data.

We describe the long-term course and pregnancy outcomes of a woman diagnosed with MPI-CDG in infancy. She was treated with oral D-mannose throughout childhood with good clinical response but demonstrated intermittent non-compliance in adulthood, complicated by hepatic fibrosis, protein-losing enteropathy, osteoporosis, anaemia, and musculoskeletal manifestations. She had two pregnancies without D-mannose supplementation.

The first pregnancy was complicated by microcytic anaemia, hypoalbuminemia, pregnancy-induced cholestasis, and emergency caesarean section. A male infant was delivered and later diagnosed with Hirschsprung disease, requiring surgical correction with good outcome. Postpartum assessment of the mother demonstrated progression of liver disease to cirrhosis (F4). The second pregnancy proceeded with fewer complications and resulted in emergency caesarean delivery of a healthy female infant with normal postnatal glucose control and growth.

This case contributes to the limited literature on pregnancy in MPI-CDG and illustrates that successful pregnancies are possible despite advanced multisystem disease and cessation of D-mannose therapy. It highlights the importance of multidisciplinary care, close hepatic and nutritional monitoring, and the need for further studies to clarify the safety and role of D-mannose supplementation during pregnancy.

## 1 Introduction

Congenital Disorder of Glycosylation Type 1b (CDG Ib; OMIM 602579), or MPI-CDG, is a deficiency of the mannose-6-phosphate isomerase enzyme. This rare autosomal recessive inherited metabolic disease is caused by a mutation in the *MPI* gene (chromosomal location: 15q24.1). The mannose-6-phosphate isomerase enzyme converts fructose-6-phosphate to mannose-6-phosphate, a key component in *N*-glycosylation. The decreased supply of mannose-6-phosphate hence results in impaired *N*-glycosylation. [1] This condition is a multi-system disorder, with common features being hepatic fibrosis, protein-losing enteropathy, hyperinsulinemic hypoglycemia, and coagulopathy. [2] Unlike other CDGs, MPI-CDG does not present with any psychomotor or mental retardation. [3] Clinical signs usually present in infancy and early childhood. Diagnosis is by either gene analysis or isoelectric focusing of transferrin. [4]

Currently, the treatment for MPI-CDG is oral D-mannose supplementation to compensate for the decreased availability of mannose-6-phosphate. [5] It is found to be an effective treatment, with good outcomes for most patients on D-mannose supplementation and poor prognosis in most patients who do not receive D-mannose. [4] No adverse effects of D-mannose supplementation have been identified, but D-mannose supplementation is still not recommended in pregnancy as its effects in pregnancy have not been sufficiently studied. [6] The use of D-mannose in pregnancy in this condition has been previously described. [7]

We are presenting a case of a woman who was diagnosed with MPI-CDG in childhood and was treated with D-mannose throughout her life until her first pregnancy at the age of 28.

## 2 Case

The diagnostic pathway [8] and the long-term follow-up [9] were previously outlined elsewhere. At 6 months, she showed mild developmental delay and failure to thrive. By 9 months, she developed hepatomegaly, and a liver biopsy revealed congenital hepatic fibrosis. She subsequently suffered from severe episodes of diarrhea and vomiting, often accompanied by hypoglycemia. Despite a small bowel biopsy showing partial villous atrophy and crypt hyperplasia, serological testing was negative for coeliac disease. Importantly, the patient had no neurological symptoms. Her antithrombin III and protein C were reduced, and her transferrin isoform pattern was consistent with MPI-CDG.

Further investigations, including skin fibroblast studies, and a thrombin tendency screen, confirmed a diagnosis of MPI-CDG. She was homozygous for a D131N (c.391G > A) variant in the *PM1* gene [9].

She was started on oral D-mannose 10 g qds (200 mg/kg/dose taken 4 times a day), which effectively managed her diarrhea, vomiting, and hypoglycemia, with improvements also seen in her thrombin screen and transferrin pattern. At age 4, she still had hepatomegaly, but her growth and development were normal.

At 18 years, non-compliance with D-mannose therapy led to the brief suspension of treatment, yet her blood results remained normal. However, by age 20, she experienced hypoglycemia, leading to two hospital admissions, as well as persistent knee pain. Bilateral knee arthroscopies revealed extensive osteochondritis dissecans, affecting multiple femoral condyles. At 21 years, she began experiencing diarrhea, abdominal pain, and low BMI, raising concerns about protein-losing enteropathy related to MPI-CDG. Despite treatment with iron infusions and supplements, she continued to suffer from fatigue.

At 22 years, she was diagnosed with coeliac disease following a positive anti-tissue transglutaminase test and duodenal biopsy, prompting a gluten-free diet. Laboratory results showed low protein C, free protein, and prolonged prothrombin time, but she remained reluctant to restart D-mannose therapy. By 23 years, she underwent another knee arthroscopy, which revealed grade III degenerative changes. Her pain was managed with codeine and paracetamol.

## 3 Preconception

Following knee surgery, the patient began physiotherapy but was admitted for bloating, abdominal pain, and frequent bowel peristalsis, which were treated as gastritis with metronidazole and ciprofloxacin. Investigations ruled out colitis, though her low hemoglobin (115 g/L) and MCV (72 fL) suggested iron deficiency anaemia and anaemia of chronic disease. Concerns arose about the progression of her MPI-CDG. Follow-up teleconsultations revealed that her gastrointestinal symptoms had improved, but intermittent abdominal pain and occult blood in stools persisted. Imaging later confirmed stage F2 liver fibrosis.

A DEXA scan at age 23 showed low bone mineral density, with *Z*-scores of −1.8 (lumbar spine), −3.3 (femoral neck), and − 2.6 (total hip), leading to a diagnosis of osteoporosis. She began cholecalciferol therapy (50,000 IU weekly for six weeks), followed by maintenance vitamin D supplementation. Dietary adjustments included a calcium-rich diet with an added glass of milk daily.

## 4 Pregnancy 1 course

At the age of 28, at 11 weeks of pregnancy, the patient reported a painful right elbow but no nausea or vomiting. Her medications, including ondansetron, were adjusted to cyclizine, and D-mannose, that was discontinued due to limited safety data. She used Polycal, and Procal as emergency products. She continued vitamin D supplementation 2000 units daily and remained under hepatology care for liver function monitoring.

At 28 weeks, she experienced sciatica, pelvic pain, and lower limb swelling, but fetal growth was normal. Her medium-term fracture risk remained low. She was on Pregnacare, cyclizine, ferrous sulfate, and Polycal. At 32 weeks, she was diagnosed with pregnancy-induced cholestasis.

Throughout the pregnancy, the patient had microcytic anaemia (Hb 94 g/L, MCV 78 fl) and hypoalbuminaemia (21 g/L). She also developed abnormal liver tests in the third trimester, with ALT 73 U/L consistent with her pregnancy-induced cholestasis. Coagulation screen done in the second trimester was normal.

**Fig. 1:**
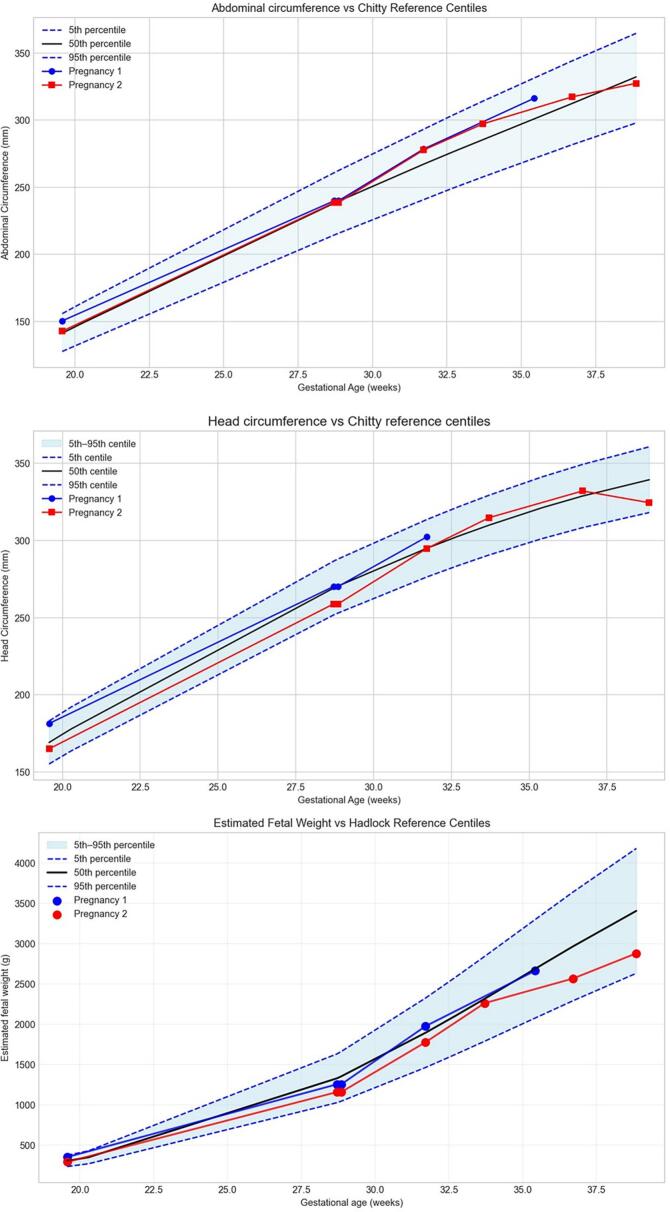
Graph of growth charts for pregnancy 1(boy) and pregnancy 2 (girl) against Chitty and Hadlock standards.

Her 20-week anomaly scan showed fetal weight above 10th centile and head and abdominal circumferences and femur length were satisfactory (Fig. 1).

### 4.1 Labour

Given the underlying metabolic condition and unclear impact on her MPI-CDG related liver disease, the patient's birth plan included induction at 38 + 4 weeks, with 10% dextrose and glucose monitoring postpartum. Capillary blood glucose was 4–6 mmol/l. She received 3 doses of prostaglandin and her membrane was ruptured during examination. Oxytocin was commenced to induce labour. Due to fetal bradycardia, she underwent an emergency C-section under spinal anesthesia.

Liver function tests showing an ALT of 129 U/L and ALP of 391 U/L (placenta-related). Her bile acids dropped from 69 to 16 umol/l. As bile acids concentration normalized and her pruritus resolved after the childbirth, abnormal liver function tests during pregnancy were attributed to obstetric cholestasis rather than liver fibrosis.

The baby boy (birth weight 2780 g; 10th centile; head circumference 35 cm; 25th centile; Apgar 9 at 1 min and 10 and 5 min) was admitted to the postnatal ward to observe his plasma glucose and lactate. While his plasma glucose has been normal since birth, his plasma lactate was initially 3–4 mmol/l and normalized. He passed meconium within the first day of life.

He was breastfed on the postnatal ward, but he was very sleepy and was not feeding well. He was being given colostrum via a syringe. On day 4 he developed some milky vomits and on day 5 he lost >12% of his birth weight (2.78 kg – > 2.44 kg). He was admitted to NICU for feeding support and his blood tests revealed acute kidney injury with serum creatinine of 144umol/L and serum urea of 17.3 mmol/L. His latest urine output is 0.66 ml/kg/h.

During an assessment of his suck reflex, he developed a bilious vomit. Additional 5 ml of bile was aspirated via his nasogastric tube. His abdomen was distended, but soft at that point. His abdominal X-ray showed gas filled loops of bowel. He was nil by mouth with intravenous fluids 150 ml/kg/day. His plasma lactate rose from 1.3 to 2.7 mmol/l.

On assessment, his abdomen was distended, loopy but soft and non-tender. Bowel sounds were active. He commenced antibiotics. A repeat abdominal X-ray confirmed gas filled bowel loops with no gas seen in the rectum. He was subsequently diagnosed with Hirschsprung disease, and successfully underwent surgery without the need for a stoma, He was readmitted for colitis which occurred three weeks later, but the baby has since had no issues and is growing normally, with no concerns regarding his development.

### 4.2 Postpartum

She was discharged home on day 6 postpartum. She required a 10-day course of flucloxacillin and tazocin to treat the wound infection.

After delivery, the patient still experienced right elbow pain and locking, attributed to osteophyte accumulation secondary to MPI-CDG. She breastfed for six months and decided not to resume D-mannose therapy. Her hemoglobin and MCV were low at 110 g/l and 78 fL, respectively, with serum iron at 5 μmol/l and saturation at 10%. Weight loss prompted dietary interventions, with recommendations to increase caloric and protein intake.

At 5 months postpartum, the patient underwent a fibroscan which revealed progression of her liver fibrosis to stage F4 (15.8 kPa). Despite this, she appeared well and reported no gastrointestinal symptoms. However, abdominal examination indicated an enlarged left lobe of the liver.

## 5 Pregnancy 2 course

Eighteen months later, she had her second pregnancy. Pregnancy developed well without any complications to labour. She had regular review by the metabolic, gastroenterology and specialist obstetrics and gynaecology review every trimester.

In the 2nd trimester she had a flare-up of her colitis with diarrhea, raised fecal calprotectin and CRP (Table 1). This responded to an increase in her mesalazine dose from 1600 mg to 4800 mg, which was subsequently reduced to a maintenance dose when her colitis symptoms settled. She continued her gluten free diet. As per dietetic advice, she had regular meals. Protein intake was estimated as 1 g/kg body weight. Hypoglycaemia was not reported, although she suffered from nausea, managed with cyclizine, in the first trimester.

**Table 1:** Biochemical and hematological results from both pregnancies.

Test + reference ranges	1st pregnancy			12 months post delivery	2nd pregnancy		
	1st trimester	2nd trimester	3rd trimester		1st trimester	2nd trimester	3rd trimester
Hb (122–160 g/L)	**110**	**106**	**119**	**110**	**113**	**109**	**117**
Platelets (150–400 × 10^9^/L)	240	184	199	382	338	215	156
Free protein S (65–115 U/dL)				89			
Protein C functional (70–140 U/dL)				120			
ALT (5–45 U/L)		15	**161**	21	23	17	40
ALP (30–130 IU/L)		54	**289**	103	93	82	**152**
Serum bilirubin (0–21 umol/l)		5	7	5	8	5	9
Albumin (35–50 g/L)		**28**	**27**	38	**34.2**	**28.9**	**27.8**
GGT (0–45 IU/L)			**272**			**63**	**97**
Bile acids (0–6 umol/L)			**63**			2	**16**
Fecal calprotectin (0–99 μg/g faec)				**>800** (15 months post-delivery)	**453**	38	
CRP (0-10 mg/ml)			6	**27** (15 months post-delivery)	9.5	8	
Plasma glucose (mmol/l)		4.3	4.8				4.7
Coagulation screen		INR 0.9 APTT 27.4 s PT 10.7 s	INR 1.1 APTT 30.4 s PT 12.6 s	Protein S 89 (65–115 (U/dL) Protein C 120 (70–140 U/dL)	INR 1.1 APTT 29 s PT 10.2 s		Protein S **0.53** (0.55–1.1) IU/L Protein C 1.14 (0.75–1.51 IU/L)
Serum iron (10.7–32.2 umol/l)			13.3	**5**	10.3	**9.2**	
Ferritin (10–180 ng/mL	42	16				47	64
IgG (6–160) IgA (0.8–2.8) IgM (0.5–2.0)				11.28 **0.22** **0.33**			

Hb- hemoglobin; INR- international normalized ratio; PT-prothrombin time; APTT- Activated Partial Thromboplastin Time; ALP-alkaline phosphatase; ALT-alanine aminotransferase; CRP- C-reactive protein; GGT- gamma-glutamyl transferase.

### 5.1 Labour

She had early signs of labour at home for 3 days before consistent and strong contractions of labour. This progressed into spontaneous labour at gestational age of 40 + 6 weeks. Entonox inhalation gas was used as pain relief during labor which lasted for 7 h without much progression. Thus, emergency caesarean section under spinal anesthetics was performed due to fetal decelerations. A baby girl was then delivered successfully with satisfactory post-partum glucose monitoring. Although a peri-delivery plan included a recommendation about the use of intravenous Dextrose 10% to treat hypoglycaemia, only Hartmann solution of 1.4 L were administered during delivery with small doses of ephedrine and phenylephrine for blood pressure support. Capillary blood glucose ranged 4–6 mmol/l.

The baby girl weighed 2960 g (7th centile) and her head circumference was 32.5 cm (9th centile). Post-delivery 24-h glucose monitoring was satisfactory. She opened her bowels and passed urine and was exclusively breastfed and there are no concerns regarding her development.

### 5.2 Post-partum

She did not have any post-partum complications and was discharged on day 2 of the post-caesarean section.

Given her previous history of osteophytes in her knees, she is awaiting evaluations of her painful elbows. There is a possibility that osteophytes are the cause of pain.

## 6 Discussion

We present a case of a woman affected with MPI-CDG, who administered D-mannose for several years in childhood and adolescence and did not find it helpful in managing her symptoms. She decided to discontinue the treatment as she found the therapy burdensome (large tablets, limited palatability, divided doses). She was aware that her colitis, osteophytes and liver disease are likely to be related to the disease progression. Shortly after she fell pregnant, she was not keen to continue the D-mannose therapy, partially due to lack of safety data in pregnancy.

In our patient, who had no past medical history of thrombi, despite a marginally deranged coagulation screen, anticoagulation was not considered during pregnancy. She was however commenced on small molecule heparin 3500 units daily for 10 days after each caesarean section. Due to her underlying liver disease, she was advised against using estradiol containing anticontraception.

Despite painful knee and elbow joints, no analgesia was used in pregnancy. Her low bone mineral density has remained stable throughout pregnancy and was managed with a high dose of vitamin D daily.

She used mesalazine at increased dose due to flare-up of her colitis. To our knowledge, this represents the first reported case of pregnancy in MPI-CDG complicated by colitis treated with mesalazine. The symptoms of bloody diarrhea and protein losing enteropathy may mimic inflammatory colitis. Ulcerative colitis as a differential diagnosis was previously excluded. With mesalazine therapy, the frequency of her flare-ups (and admissions to hospital) was significantly reduced before pregnancies.

There have been few case reports involving pregnancy in MPI-CDG. One case describes a patient who initially presented with gastrointestinal symptoms and hypercoagulability during childhood. Although treated with anticoagulation, the diagnosis of MPI-CDG was not established until adulthood. By the time of diagnosis, she had already experienced three uneventful pregnancies, resulting in the birth of healthy children. Notably, she had an older brother who presented with similar symptoms to her and died at age 5 from gastrointestinal bleeding. [10]

Another case describes a patient who presented with hypoglycemia and hepatomegaly as a child and was diagnosed with MPI-CDG at age 6. Although started on D-mannose therapy, she was noncompliant with it. In adulthood, she began experiencing recurrent episodes of deep vein thromboses (DVTs). Upon discovering her pregnancy at seven weeks, she commenced a brief course of apixaban and D-mannose, both of which were discontinued due to her pregnancy. Complications during her pregnancy included a 20 kg weight gain, pyelonephritis, and proteinuria without preeclampsia in the third trimester. She then had a C-section at 41 weeks due to cervical dystocia. Her child was breastfed and had no issues as of 2 years old. [11]

The last case involves an asymptomatic patient who had two children with no reported complications apart from test finding decreased levels of protein C, free protein S, and antithrombin III activity. [12]

A recent case of a 26-year-old French woman described her experience of D-mannose use in pregnancy. It not only helped manage her hypoglycaemia and improved biochemical parameters, but also proved to be safe for the baby [7].

Despite discontinuation of D-mannose, a favorable maternal and fetal outcome is achievable in MPI-CDG complicated by significant baseline autoimmune disease through coordinated multidisciplinary management, proactive metabolic optimization and careful anticipation of hematological and biochemical, and obstetric complications.

## Author contribution

JHML, MOK, JB- manuscript draft; AH, AH, AM, SJ, SM, AW, PS- clinical expertise; KMS- concept, clinical expertise.

## CRediT authorship contribution statement

**Jia Hui Megan Loh:** Writing – review & editing, Writing – original draft, Methodology, Investigation, Data curation. **May Oo Khin:** Writing – review & editing, Writing – original draft, Visualization, Validation, Resources, Investigation, Data curation. **John Bassett:** Writing – review & editing, Writing – original draft, Visualization, Validation, Investigation, Formal analysis, Data curation. **Amal Mighell:** Resources, Investigation. **Sarah Jowett:** Writing – original draft, Resources, Methodology, Investigation, Data curation. **Sulleman Moreea:** Investigation, Methodology. **Alison Woodall:** Resources, Investigation. **Priscilla Sia:** Resources, Investigation. **Afifah Hussain:** Writing – original draft, Resources, Methodology, Investigation. **Amy Hufton:** Writing – original draft, Validation, Supervision, Conceptualization. **Karolina M. Stepien:** Writing – review & editing, Writing – original draft, Validation, Supervision, Resources, Project administration, Methodology, Investigation, Formal analysis, Data curation, Conceptualization.

## Consent

Written consent was obtained from the patient.

## Guarantor

KMS.

## Funding

None.

## Declaration of competing interest

None.

## Data Availability

Data will be made available on request.
